# The impact of sleep duration on the incidence of new-onset chronic kidney disease in community-dwelling adults: a nationwide cohort study

**DOI:** 10.1080/07853890.2025.2569986

**Published:** 2025-10-27

**Authors:** Jung-Hwan Kim, Min-Jin Ha, Dong-Woo Choi, Sohee Park

**Affiliations:** ^a^ Department of Health Informatics and Biostatistics, Graduate School of Public Health, Yonsei University, Seoul, Republic of Korea; ^b^ Department of Family Medicine, Severance Hospital, Yonsei University College of Medicine, Seoul, Republic of Korea; ^c^ Division of Biohealth Data Science, Graduate School of Transdisciplinary Health Sciences, Yonsei University, Seoul, Republic of Korea; ^d^ Cancer Big Data Center, National Cancer Center, Goyang-si, Republic of Korea

**Keywords:** Sleep length, CKD, cohort, Korea

## Abstract

**Background:**

This research examined the association between sleep duration and new-onset chronic kidney disease (CKD) among community-dwelling middle-aged and elderly individuals in Korea.

**Methods:**

This prospective cohort study utilized data from the Korean Genome and Epidemiology Study (KoGES) from 2001–2002 (baseline) to 2019–2020 (tenth follow-up visit). New-onset CKD was the primary outcome, defined as an estimated glomerular filtration rate <60 mL/min/1.73 m^2^ or the proteinuria. Study populations were classified into six self-reported sleep length categories: <5h, 5h ≤ to <6h, 6h ≤ to <7h, 7h ≤ to ≤8h, 8h < to <9h, and 9h/day ≤. Cox proportional hazards models were used to ascertain the hazard ratios (HRs) and 95% confidence intervals (CIs) for CKD incidence across these categories.

**Results:**

Over a median follow-up duration of 17.41 years, CKD was identified in 551 (14.4%) of 3835 participants (mean age 48.7 ± 7.5 years). After adjusting for confounding variables, a U-shaped relationship between sleep lengths and CKD was identified. Participants with insufficient (<5h) and excessive (9h ≤) sleep length exhibited HRs for CKD incidence of 1.44 (1.09–1.89) and 1.85 (1.04–3.27), respectively, compared to individuals with normal sleep length (7h ≤ to ≤8h). Age and sex differences were observed in the association between sleep length and CKD incidence. The association between sleep duration and new-onset CKD was significant only in participants aged 40 to 64 years, with no significant association observed in individuals aged 65 years and older.

**Conclusions:**

This research identified a relationship between the amount of sleep and CKD in Korean adults. Maintaining an appropriate sleep duration of 7–8 h/day is important for preventing new-onset CKD.

## KEY MESSAGES

- The association between sleep duration and new-onset CKD in the Korean population is still unclear.
- Both insufficient and excessive sleep lengths were associated with a higher risk of incident CKD.
- Age and sex differences were observed in the association between sleep length and CKD incidence.

## Graphical Abstract

**Figure:**
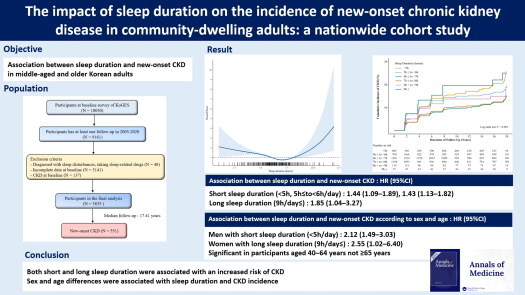

## 1. Introduction

Chronic kidney disease (CKD) poses a critical global health concern, characterized by an ongoing deterioration in kidney function, which results in an increased prevalence of the disease and deaths [1]. In 2017, it was reported that the global prevalence of CKD stood at around 9.1% [2], while, the prevalence in Korea was reported to be 8.2% [3]. By 2040, CKD is anticipated to become the fifth leading cause of years of life lost worldwide [4]. From 2008 to 2011, the financial burden associated with kidney diseases in affected individuals increased from 899 million dollars to 1.43 billion dollars [5]. Several health organizations have highlighted the importance of initial prevention for CKD. Individuals are advised to concentrate on minimizing modifiable risk factors, which include lack of physical activity, obesity, and poor dietary patterns, tobacco use, and alcohol intake [6].

Proper sleep is crucial for maintaining overall health, as it significantly contributes to physical wellness and improves general well-being [7]. Although the required amount of sleep can vary across different life stages, the National Sleep Foundation (NSF) advises that individuals in their middle age should target 7 to 8 h of nightly rest [8]. Sleep length may affect health in multiple ways. Findings indicate that both inadequate and excessive sleep lengths are linked to a heightened likelihood of developing cardiovascular disease (CVD) [9], overall mortality [10], metabolic syndrome [11], involving high blood sugar [12], high blood pressure [13], and overweight [14]. All these factors contribute to the likelihood of CKD. Thus, it is suggested that sleep may be connected to CKD; however, additional research is needed to establish whether this connection exists independently of comorbidities and other important confounding elements including overweight, insufficient exercise, alcohol intake, and tobacco use. Prior research exploring the link between the amount of sleep and CKD has produced differing findings. Furthermore, there is an absence of extensive longitudinal cohort research that examines the impact of sleep length and the likelihood of developing CKD in adults living in the community in Korea. Consequently, this research focused on examining how sleep duration relates to the occurrence of new cases of CKD in middle-aged and elderly individuals residing in the community in Korea, based on information from a substantial longitudinal cohort investigation.

## 2. Materials and methods

### 2.1. Participants

**Figure 1.**
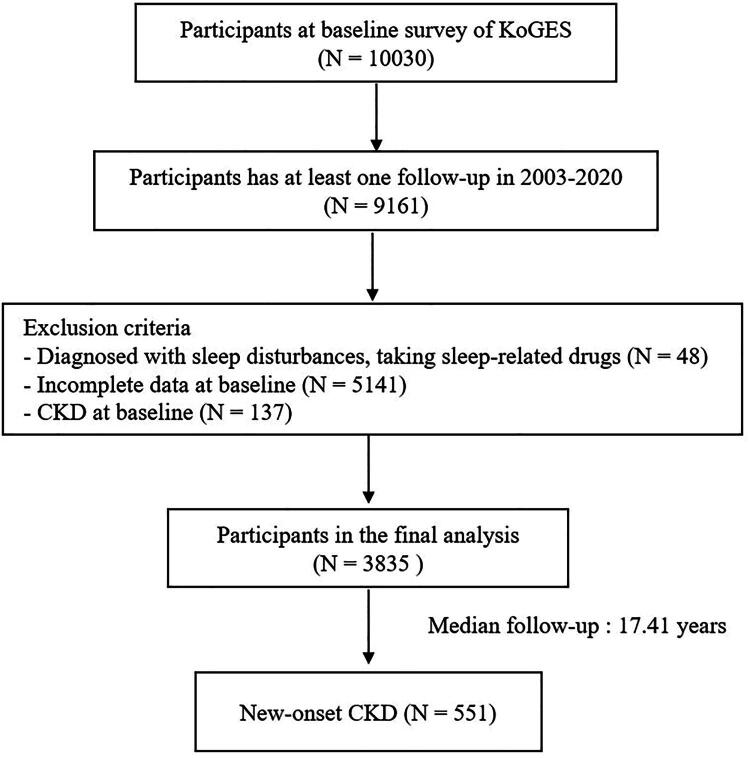
Flowchart of the study population.

This research examined data from the Korean Genome and Epidemiology Study (KoGES), which is managed by the National Institute of Health at the Korea Disease Control and Prevention Agency (KDCA) to determine how environmental and genetic factors influence the risk of non-communicable diseases. The KoGES-Ansan and Anseong study is a community-oriented, prospective, longitudinal cohort investigation. This cohort study included 10030 participants (aged 40–69) living in urban (Ansan) or rural (Anseong) areas. It was conducted biennially from a baseline in 2001–2002 through the tenth follow-up in 2019–2020. For baseline assessment, eligible individuals were initially recruited through on-site invitations and postal correspondence requesting their voluntary participation. Subsequently, respondents were directed to one of over 50 national and international medical schools, hospitals, and healthcare institutions. Written informed consent for participation in the KoGES cohort was obtained from all study participants at the time of their enrolment [15]. However, the authors of this current study did not directly obtain individual informed consent from participants. The current study protocol was granted approval by the Institutional Review Board (IRB) at Severance Hospital (Approval number 4-2024-0717). To assess the development of new-onset CKD, the study excluded 48 participants diagnosed with sleep disturbances and who were using sleep-related medications, 137 individuals with CKD at baseline (defined as an eGFR <60 mL/min/1.73m^2^ or proteinuria ≥1+), and 5141 participants with incomplete data on confounding variables. Ultimately, the final evaluation comprised 3835 individuals (Figure 1).

### 2.2. Anthropometric and laboratory assessments

The weights and heights of the subjects were recorded while they were wearing lightweight indoor garments and without footwear, with measurements obtained to an accuracy of 0.1 kg for mass and 0.1 cm for stature. The body mass index (BMI) is determined by dividing an individual’s weight in kilograms by the square of their height in metres, a widely accepted indicator of obesity, as recognized by the World Health Organization (WHO) [16]. Waist circumference (WC) was assessed after a normal exhalation, at a location equidistant from the lower edge of the thoracic cavity and the pelvic brim, ensuring alignment with the horizontal plane, with a precision of 0.1 cm. For blood pressure assessment, participants were seated, and measurements were taken twice at one-minute intervals using a mercurial sphygmomanometer, adhering to an established protocol.

Blood specimens were collected from the antecubital vein following a fasting period of 12 h. C-reactive protein (CRP) levels were evaluated using a Roche/Hitachi 912 System (Roche Diagnostics, Indianapolis, IN, USA) *via* the latex-enhanced immunoturbidimetric approach, with a detection threshold of 0.09 mg/L. Creatinine, Fasting plasma glucose (FPG), glycated haemoglobin (HbA1c), total cholesterol, low-density lipoprotein cholesterol (LDL-C), high-density lipoprotein cholesterol (HDL-C), and triglyceride levels were assessed using enzymatic techniques with a biochemical analyser (Hitachi 7600; Hitachi Co., Tokyo, Japan).

The self-reported questionnaires collected data on degree of physical activity, tobacco use, and alcohol intake. The degree of physical activity was evaluated through metabolic equivalents of task (MET), which reflect the amount of energy expended associated with an activity in comparison to resting levels, calculated based on the overall average hours of activity each week. Non-smoker was defined as a person who had never smoked at any point in their lives, whereas smoker was defined as a person who had smoked at any time in the past or indicated current smoking behaviours in the survey. Alcohol consumption was defined as excessive if it exceeded 10 grams daily for women and 20 grams daily for men. Hypertension (HTN) was defined by a systolic blood pressure (SBP) ≥140 mmHg, a diastolic blood pressure (DBP) ≥90 mmHg, or the ongoing use of antihypertensive drugs [17]. DM is defined by one or more of the following standards: a FPG level ≥126 mg/dL, a plasma glucose concentration ≥200 mg/dL measured two hours after administering a 75 g oral glucose tolerance test, a HbA1c level ≥6.5%, a diagnosis made by a medical professional, or the regular use of anti-diabetic drugs [18]. Comprehensive study protocols can be found at: https://nih.go.kr/ko/main/contents.do?menuNo=300563

### 2.3. Definition of variables

#### 2.3.1. Definition of chronic kidney disease (CKD)

The primary outcome was the incidence of newly diagnosed CKD, characterized by either the detection of proteinuria, as shown by a dipstick urine analysis with a protein concentration of 1+ or higher or an estimated glomerular filtration rate (eGFR) <60 mL/min/1.73 m^2^. The eGFR was derived following the CKD Epidemiology Collaboration (CKD-EPI) formula [19].

The CKD-EPI formula is expressed in the following manner:

$$\text{eGFR}=141\times \text{min}{\left(\text{Scr}/\kappa ,\;1\right)}^{\alpha }\times \text{max}{\left(\text{Scr}/\kappa ,\;1\right)}^{-1.209}\times 0.993^{\text{Age}}\times 1.018\left[\text{if women}\right]$$

*where k* is 0.9 for men and 0.7 for women, *α* = −0.411 for men and −0.329 for women, *Scr* is serum creatinine (mg/dL), max denotes the maximum of *Scr*/*k* or 1 and min denotes the minimum of *Scr*/*k* or 1. The definition of new-onset CKD indicates CKD diagnosed during the follow-up period in individuals who did not have CKD prior to this period.

#### 2.3.2. Definition of sleep duration

The National Sleep Foundation (NSF) advises that individuals aged 18 to 64 should strive for 7 to 9 h of sleep each night, whereas seniors aged 65 and above ought to focus on obtaining 7 to 8 h [20]. Furthermore, American Academy of Sleep Medicine (AASM) and the Sleep Research Society (SRS) advise that adults aged 18 to 60 are encouraged to aim to strive for a minimum of 7 h of sleep each night on a consistent basis [21]. Drawing upon these recommendations which suggest optimal sleep durations, and informed by previous research [22] that adopted similar classification methodologies, participants were classified into six categories based on their self-reported sleep length: <5h, 5h ≤ to <6h, 6h ≤ to <7h, 7h ≤ to ≤8h, 8h < to <9h, and 9h/day ≤. This detailed categorization aims to capture potential differential risks associated with varying degrees of sleep duration and to better elucidate the dose-response relationship.

### 2.4. Statistical analysis

To evaluate the clinical features of the participants, the chi-squared test was employed for categorical variables, whereas analysis of variance (ANOVA) was employed for continuous variables. Continuous data are presented as the average ± standard deviation (SD), while categorical data are described as counts (percentage; %). Cox proportional hazards regression analysis was carried out to examine the correlation between the development of CKD across different categories of sleep duration. The hazard ratios (HR) along with their associated 95% confidence intervals (CIs) were determined for the incidence of new-onset CKD. Three models were developed for this analysis: Model 1 was unadjusted; Model 2 was adjusted for age and sex; and Model 3 was adjusted for age, sex, BMI, alcohol intake, smoking status, physical activity, DM, HTN, and CRP. Covariates were selected based on criteria encompassing empirical evidence from previous large-scale epidemiological studies [23,24] that identified them as risk factors for CKD, and their clinical significance as potential confounders in the relationship between sleep duration and renal outcomes. To examine the cumulative incidence rates of newly diagnosed CKD among various categories of sleep duration, Kaplan–Meier plots and the log-rank test were utilized. The proportional hazards assumption for all Cox proportional hazards models was visually inspected using Kaplan-Meier curves and formally tested using Schoenfeld residuals. No significant violations of the proportional hazards assumption (*p* > 0.05) were observed for any of the covariates included in the final models, indicating the appropriateness of the Cox regression approach. To prevent reverse causality, a sensitivity analysis was conducted by Cox proportional hazards models on a dataset that excluded all participants who developed new-onset CKD within the first two years of follow-up from the baseline assessment, thereby evaluating the consistency of the association between sleep duration and CKD incidence. A subgroup analysis was carried out to examine the correlation between sleep duration and the incidence of new-onset CKD within various subgroups categorized by age, sex, and comorbid conditions. A criterion for statistical significance set at *p* < 0.05. The analyses used in the research were conducted using R 4.3.0 and SAS 9.4, with both software provided by R Foundation for Statistical Computing, Vienna, Austria, and SAS Institute Inc., Cary, North Carolina, USA, respectively.

## 3. Results

### 3.1. Baseline characteristics of participants based on sleep duration

Table 1 displays the baseline features of the participants categorized by sleep length. Among the 3835 participants with a mean age of 48.7 ± 7.5 years, a total of 551 participants (14.4%) experienced the newly diagnosed CKD over a median follow-up duration of 17.41 years following the conclusion of the ninth follow-up assessment. The occurrence of CKD was significantly higher in individuals with short (<5h, 5h ≤ to <6h) and long (9h ≤) sleep durations compared to the usual (7h ≤ to ≤8h) sleep length. Participants with short sleep length (<5h) and long sleep length (9h ≤) were more likely to have a higher BMI (*p* = 0.011) and SBP (*p* = 0.005) compared with those with normal sleep length (7h ≤ to ≤8h).

**Table 1.** Baseline characteristics of the study population according to the sleep duration.

Variables^a^	Overall^b^	<5h	5h ≤ to <6h	6h ≤ to <7h	7h ≤ to ≤8h	8h < to <9h	9h ≤	*p*-value
Numbers, *n*	3,835	406	760	1,301	1,194	119	55	
Age, years	48.7 ± 7.5	50.5 ± 8.3	49.0 ± 7.7	48.1 ± 7.2	48.6 ± 7.4	47.2 ± 6.9	48.5 ± 7.5	<0.001
Sex								<0.001
Men	1,985 (51.8%)	171 (42.1%)	334 (43.9%)	683 (52.5%)	703 (58.9%)	59 (49.6%)	35 (63.6%)	
Women	1,850 (48.2%)	235 (57.9%)	426 (56.1%)	618 (47.5%)	491 (41.1%)	60 (50.4%)	20 (36.4%)	
BMI, kg/m^2^	24.7 ± 2.9	25.2 ± 3.1	24.9 ± 3.0	24.6 ± 2.9	24.6 ± 2.9	24.5 ± 3.3	24.6 ± 3.1	0.011
WC, cm	80.9 ± 8.3	81.4 ± 8.3	80.5 ± 8.1	80.8 ± 8.5	81.0 ± 8.2	80.0 ± 9.0	81.6 ± 9.7	0.411
SBP, mmHg	116.5 ± 16.9	118.4 ± 18.2	116.8 ± 16.6	115.4 ± 16.2	117.2 ± 17.0	113.4 ± 16.3	118.1 ± 21.8	0.005
DBP, mmHg	78.0 ± 11.4	78.5 ± 11.8	78.0 ± 11.2	77.5 ± 11.3	78.3 ± 11.3	76.7 ± 10.6	78.9 ± 13.6	0.312
FPG, mg/dL	88.0 ± 21.8	88.9 ± 24.4	87.1 ± 19.5	87.8 ± 22.2	88.5 ± 21.7	88.7 ± 22.4	88.5 ± 22.5	0.715
HbA1c, %	5.7 ± 0.8	5.8 ± 1.0	5.7 ± 0.8	5.7 ± 0.8	5.7 ± 0.8	5.7 ± 0.6	5.7 ± 0.8	0.565
Creatinine, mg/dL	0.9 ± 0.2	0.8 ± 0.2	0.8 ± 0.2	0.9 ± 0.2	0.9 ± 0.2	0.9 ± 0.2	0.9 ± 0.2	<0.001
Total cholesterol, mg/dL	196.1 ± 34.9	197.4 ± 34.9	197.1 ± 33.9	195.8 ± 34.8	195.8 ± 35.2	194.4 ± 40.9	190.8 ± 31.6	0.707
Triglyceride, mg/dL	157.3 ± 98.3	157.0 ± 102.4	159.4 ± 93.8	156.7 ± 102.5	156.2 ± 90.2	151.3 ± 81.2	183.2 ± 190.0	0.444
HDL-cholesterol, mg/dL	44.8 ± 9.7	44.9 ± 9.9	44.9 ± 9.9	45.0 ± 9.7	44.4 ± 9.4	45.4 ± 10.6	42.7 ± 8.7	0.28
LDL-cholesterol, mg/dL	119.9 ± 32.5	121.2 ± 34.0	120.3 ± 32.0	119.5 ± 31.7	120.2 ± 32.8	118.7 ± 32.8	111.5 ± 42.7	0.424
CRP, mg/dL	0.2 ± 0.4	0.2 ± 0.4	0.2 ± 0.3	0.2 ± 0.3	0.2 ± 0.4	0.2 ± 0.2	0.2 ± 0.3	0.306
Alcohol intake, n (%)	2,020 (52.7%)	173 (42.6%)	368 (48.4%)	701 (53.9%)	678 (56.8%)	69 (58.0%)	31 (56.4%)	<0.001
Smoking, n (%)	914 (23.8%)	79 (19.5%)	160 (21.1%)	284 (21.8%)	345 (28.9%)	33 (27.7%)	13 (23.6%)	<0.001
Physical activity, METs	126.1 ± 66.6	125.8 ± 69.0	128.9 ± 66.1	128.4 ± 68.3	121.5 ± 63.5	134.3 ± 72.8	114.2 ± 65.7	0.036
DM, *n* (%)	453 (11.8%)	58 (14.3%)	82 (10.8%)	147 (11.3%)	142 (11.9%)	13 (10.9%)	11 (20.0%)	0.212
HTN, *n* (%)	894 (23.3%)	115 (28.3%)	185 (24.3%)	282 (21.7%)	280 (23.5%)	23 (19.3%)	9 (16.4%)	0.059
CKD, *n* (%)	551 (14.4%)	84 (20.7%)	129 (17.0%)	164 (12.6%)	144 (12.1%)	17 (14.3%)	13 (23.6%)	<0.001
Sleep duration, hours	6.3 ± 1.2	4.1 ± 0.6	5.3 ± 0.3	6.3 ± 0.3	7.4 ± 0.4	8.5 ± 0.2	9.4 ± 0.7	<0.001

^a^ Data are expressed as mean ± standard deviation for continuous variables and number (percentage) for categorical variables.

^b^ Groups determined by sleep duration (hours).

BMI: Body mass index; WC: Waist circumference; SBP: Systolic blood pressure; DBP: Diastolic blood pressure; FPG: Fasting plasma glucose; HbA1c: Glycated haemoglobin; HDL: high-density lipoprotein; LDL: low density lipoprotein; CRP: C-reactive protein; METs: Metabolic equivalents; DM: Diabetes mellitus; HTN: Hypertension; CKD: Chronic kidney disease.

Table 2 presents the occurrence of CKD throughout the follow-up intervals. The evaluation of newly developed CKD was performed every two years. The peak biennial incidence rate of newly diagnosed CKD was observed during 2003–2004, reaching 4.5, which then decreased to a low of 0.4 in 2011–2012, before rising again to 2.4 by the period of 2019–2020.

**Table 2.** Incidence of new-onset CKD during the follow up study.

Year range	Follow-up	Total numbers, *n*	Incident cases, *n*	Incident rate per 2 years, %
2001–2002	Baseline	3,835		
2003–2004	2 years	3,835	171	4.5
2005–2006	4 years	3,226	122	3.8
2007–2008	6 years	2,998	65	2.2
2009–2010	8 years	2,932	51	1.7
2011–2012	10 years	2,739	12	0.4
2013–2014	12 years	2,694	15	0.6
2015–2016	14 years	2,746	23	0.8
2017–2018	16 years	2,632	30	1.1
2019–2020	18 years	2,543	62	2.4

### 3.2. Relationship between sleep duration and new-onset CKD

Table 3 displays the findings from the Cox proportional hazards regression analyses concerning the occurrence of newly developed CKD in relation to sleep length. In the unadjusted model, the HRs for new-onset CKD associated with short sleep lengths (<5h and 5h ≤ to <6h) and long sleep lengths (9h ≤) were 1.77 (1.35–2.31), 1.40 (1.11–1.78), and 2.00 (1.14–3.54), respectively, when compared to those with a normal sleep length (7h ≤ to ≤8h). In Model 2, the HRs were adjusted to 1.44 (1.09–1.88), 1.39 (1.09–1.76), and 1.97 (1.12–3.49), respectively. For Model 3, the HRs were further adjusted to 1.44 (1.09–1.89), 1.43 (1.13–1.82), and 1.85 (1.04–3.27), respectively.

**Table 3.** Cox proportional hazard regression analysis for incident CKD based on the sleep duration.

CKD	<5h		5h ≤ to <6h		6h ≤ to <7h		7h ≤ to ≤8h		8h < to <9h		9h ≤	
	HR (95% CI)	*p*-value	HR (95% CI)	*p*-value	HR (95% CI)	*p*-value	HR (95% CI)	*p*-value	HR (95% CI)	*p*-value	HR (95% CI)	*p*-value
Model 1^a^	1.77 (1.35–2.31)	<0.001	1.40 (1.11–1.78)	0.005	1.01 (0.80–1.26)	0.963	1	ref	1.24 (0.75–2.05)	0.402	2.00 (1.14–3.54)	0.016
Model 2^b^	1.44 (1.09–1.88)	0.009	1.39 (1.09–1.76)	0.007	1.05 (0.84–1.31)	0.684	1	ref	1.39 (0.84–2.31)	0.196	1.97 (1.12–3.49)	0.019
Model 3^c^	1.44 (1.09–1.89)	0.010	1.43 (1.13–1.82)	0.003	1.05 (0.84–1.32)	0.671	1	ref	1.33 (0.78–2.26)	0.299	1.85 (1.04–3.27)	0.036

^a^ Unadjusted model.

^b^ Adjusted for age and sex.

^c^ Adjusted for age, sex, BMI, alcohol intake, smoking, physical activity, DM, HTN, and CRP.

Groups determined by sleep duration (hours).

CKD: Chronic kidney disease; HR: Hazard ratio; CI: Confidence interval; BMI: Body mass index; DM: Diabetes mellitus; HTN: Hypertension; CRP: C-reactive protein.

### 3.3. Sensitivity analysis

The findings from the sensitivity analysis, after excluding incident CKD cases that occurred within the first two years of follow-up (*n* = 161 exclusions), demonstrated the persistence of the U-shaped association between sleep duration and CKD incidence. The HRs for unhealthy sleep durations (short and long) remained largely consistent with the primary analysis, suggesting that the observed relationships were robust against reverse causality (Table 4).

**Table 4.** Cox proportional hazard regression analysis for incident CKD based on the sleep duration excluding those who developed new-onset CKD within 2 years of follow-up from the baseline assessment.

CKD	<5h		5h ≤ to <6h		6h ≤ to <7h		7h ≤ to ≤8h		8h < to <9h		9h ≤	
	HR (95% CI)	*p*-value	HR (95% CI)	*p*-value	HR (95% CI)	*p*-value	HR (95% CI)	*p*-value	HR (95% CI)	*p*-value	HR (95% CI)	*p*-value
Model 1^a^	1.90 (1.39–2.59)	<0.001	1.14 (0.85–1.53)	0.382	1.02 (0.79–1.33)	0.866	1	ref	1.13 (0.61–2.10)	0.699	1.91 (0.97–3.78)	0.062
Model 2^b^	1.66 (1.21–2.27)	0.002	1.18 (0.88–1.59)	0.262	1.08 (0.83–1.40)	0.578	1	ref	1.26 (0.68–2.35)	0.459	1.85 (0.93–3.65)	0.078
Model 3^c^	1.65 (1.20–2.26)	0.002	1.21 (0.90–1.63)	0.202	1.06 (0.82–1.38)	0.648	1	ref	1.33 (0.72–2.49)	0.364	1.69 (0.85–3.34)	0.135

^a^ Unadjusted model.

^b^ Adjusted for age and sex.

^c^ Adjusted for age, sex, BMI, alcohol intake, smoking, and physical activity, DM, HTN, and CRP.

Groups determined by sleep duration (hours).

CKD: Chronic kidney disease; HR: Hazard ratio; CI: Confidence interval; BMI: Body mass index; DM: Diabetes mellitus; HTN: Hypertension; CRP: C-reactive protein.

### 3.4. Association between sleep duration and new-onset CKD according to age and sex

Figure 2A illustrates the HRs along with 95% CIs of the occurrence of newly diagnosed CKD based on sleep length categories, analysed according to age and sex. In the age subgroup analysis within Model 3, compared to those with normal sleep length (7h ≤ to ≤8h), the HRs (with 95% CIs) for participants aged 40–64 years with short sleep lengths (<5h and 5h ≤ to <6h) and long sleep length (9h ≤) were 1.69 (1.26–2.26), 1.48 (1.14–1.91), and 1.90 (1.02–3.53), respectively. Individuals aged 65 years and older did not exhibit a notable difference in the risk of new-onset CKD across the various sleep length categories. In Model 3, when compared to individuals with normal sleep length (7h ≤ to ≤8h), the HR (95% CIs) of new-onset CKD in males with short sleep length (<5h) was 2.12 (1.49–3.03). For females with a long sleep length (9h ≤), the HR was 2.55 (1.02–6.40).

Figure 2.A. Forest Plot of the cox proportional HRs and 95% CIs for incident CKD based on sleep duration categorized by sex and age. B. Forest Plot of the cox proportional HRs and 95% CIs for incident CKD based on sleep duration categorized by comorbidities.

**Figure:**
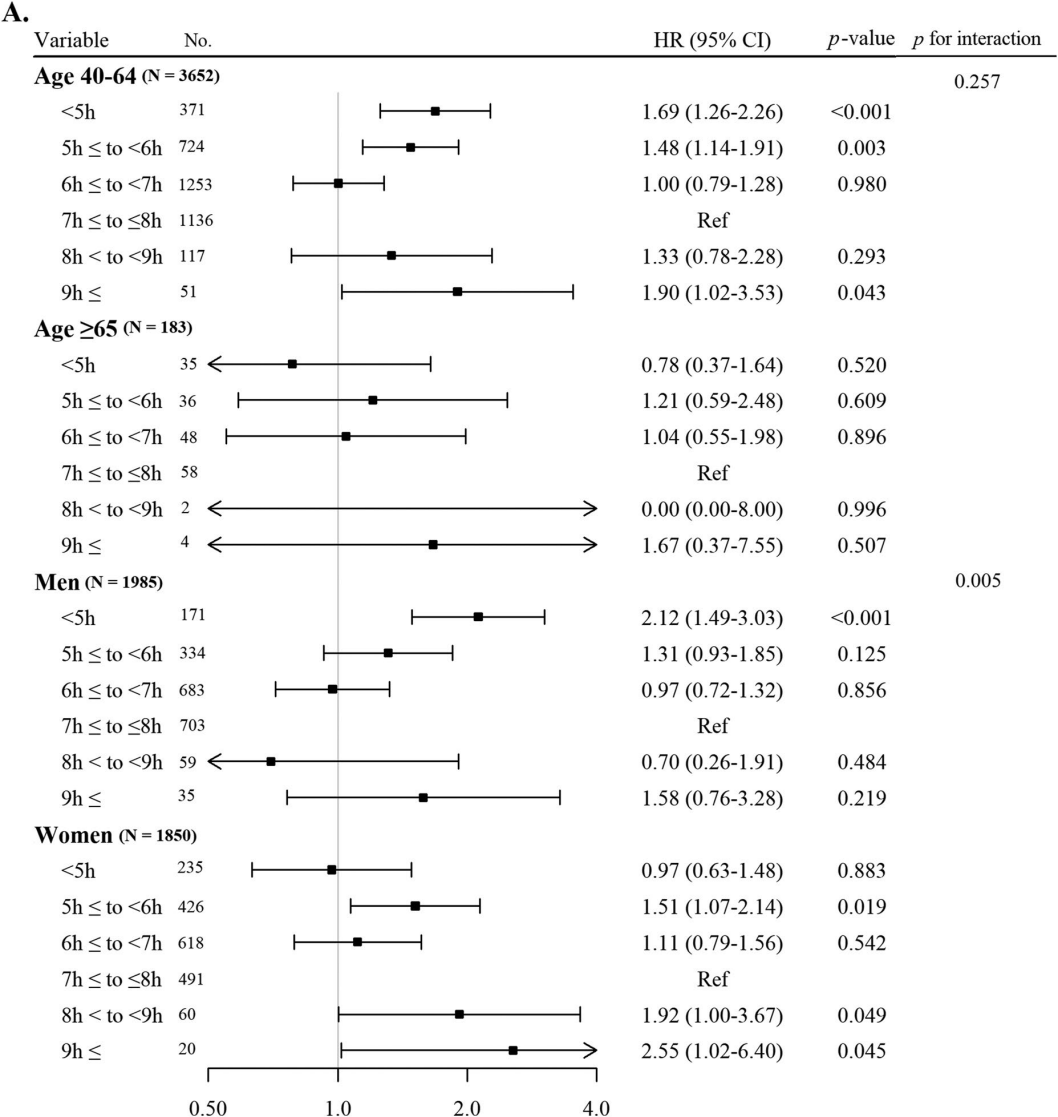

**Figure:**
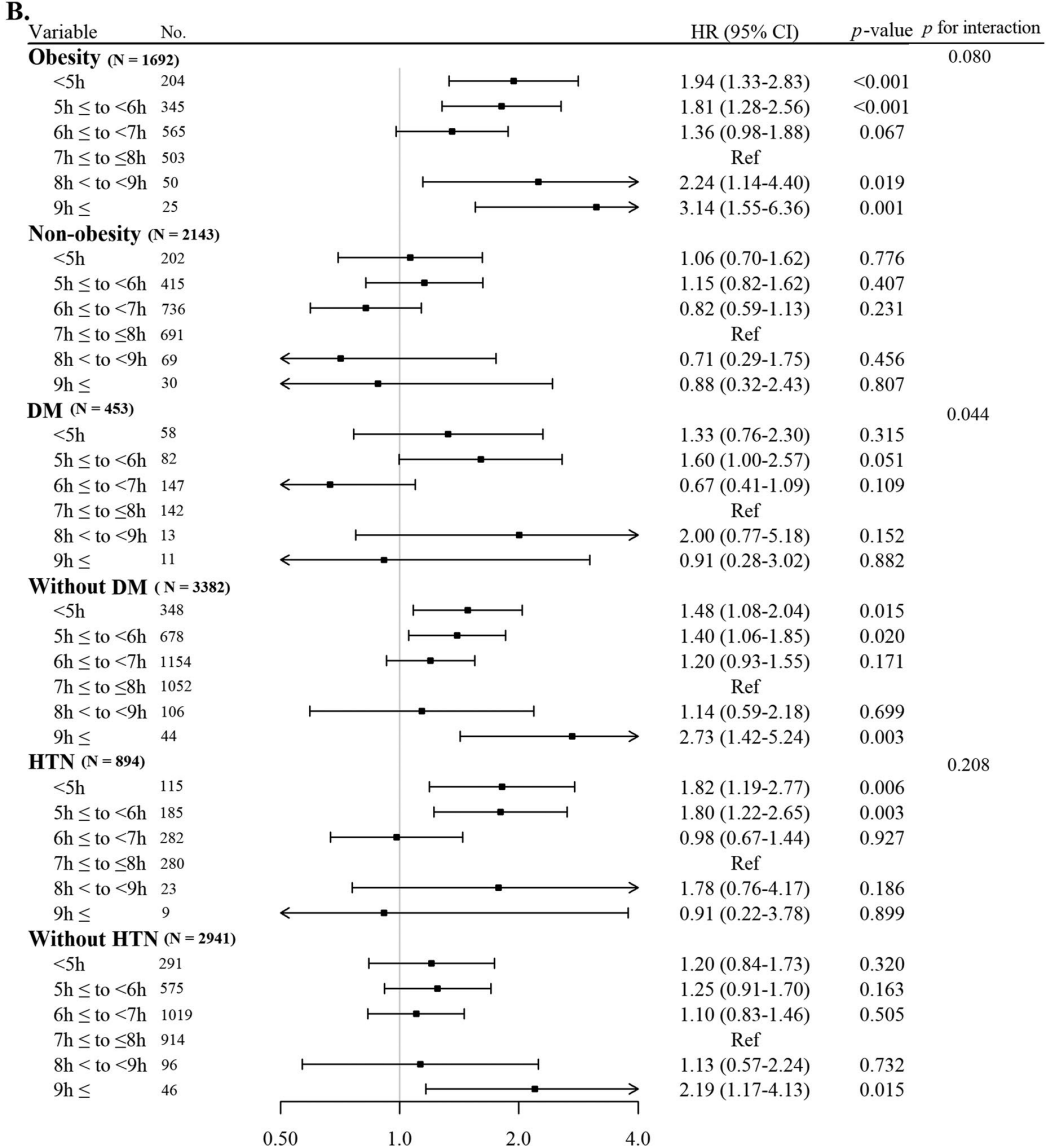

### 3.5. Association between sleep duration and new-onset CKD according to comorbidities

Figure 2B presents the HRs along with 95% CIs of the incidence of newly diagnosed CKD categorized by sleep length in a subgroup analysis based on comorbidities. In Model 3, after adjusting for various confounders except for obesity, compared to those with normal sleep length (7h ≤ to ≤8h), the HRs (95% CIs) for newly diagnosed CKD for individuals with obesity and short sleep lengths (<5h and 5h ≤ to <6h) as well as for long sleep lengths (8h < to <9h, 9h ≤) were 1.94 (1.33–2.83), 1.81 (1.28–1.56), 2.24 (1.14–4.40), and 3.14 (1.55–6.36), respectively. In contrast, participants who were not obese showed no notable variation in the incidence of newly diagnosed CKD based on sleep length. In the subgroup evaluation concerning the existence of DM, after adjusting for other confounders in Model 3 except for DM, the HRs (95% CIs) of new-onset CKD among participants without DM but with short sleep lengths (<5h and 5h ≤ to <6h) and long sleep lengths (9h ≤) were 1.48 (1.08–2.04), 1.40 (1.06–1.85), and 2.73 (1.42–5.24), respectively. No significant difference was found in the likelihood of newly diagnosed CKD among those with DM based on sleep length. For participants with HTN, after adjusting for other confounders in Model 3 except for HTN, the HRs for those with short sleep lengths (<5h and 5h ≤ to <6h) were 1.82 (1.19–2.77) and 1.80 (1.22–2.65), respectively. For individuals without HTN but with long sleep length (9h ≤), the HR was 2.19 (1.17–4.13). Among participants without HTN, the HR for long sleep length (9h ≤) was also 2.19 (1.17–4.13).

### 3.6. Cumulative incidence of CKD according to sleep duration

**Figure 3.**
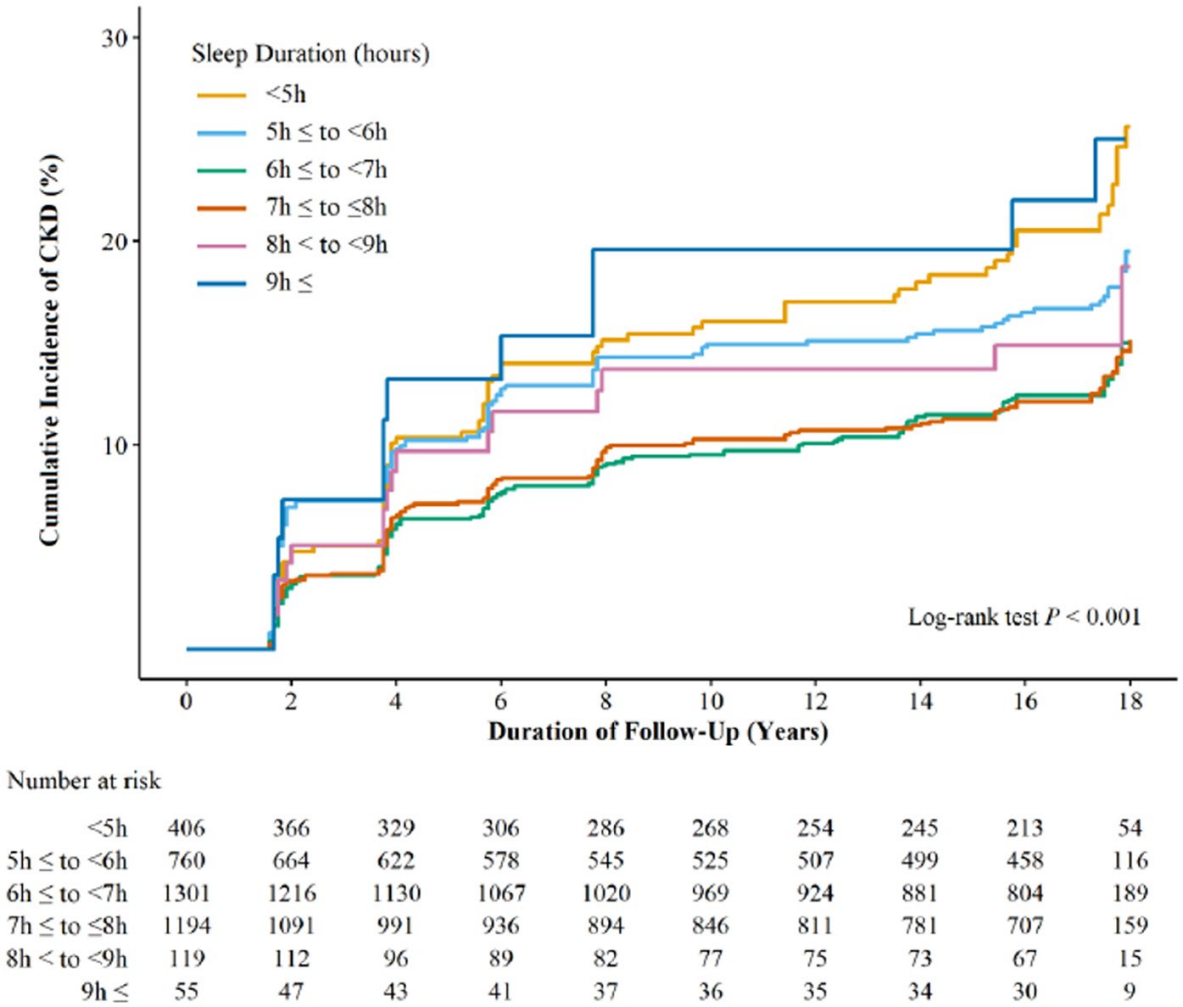
Kaplan–Meier curves displaying cumulative incident CKD based on sleep duration.

Figure 3 illustrates the Kaplan–Meier curves along with the results of the log-rank test that show the cumulative incidence of CKD in relation to sleep length. Individuals who reported short sleep lengths (<5h and 5h ≤ to <6h) and those with long sleep lengths (8h < to < 9h and 9h ≤) presented a significant relationship with an increased occurrence of CKD in comparison to individuals with usual sleep length (7h ≤ to ≤8h) at each examination visit throughout the duration of follow-up (log-rank test *p* < 0.001).

### 3.7. Cox proportional hazards spline curves for CKD incidence based on sleep duration

**Figure 4.**
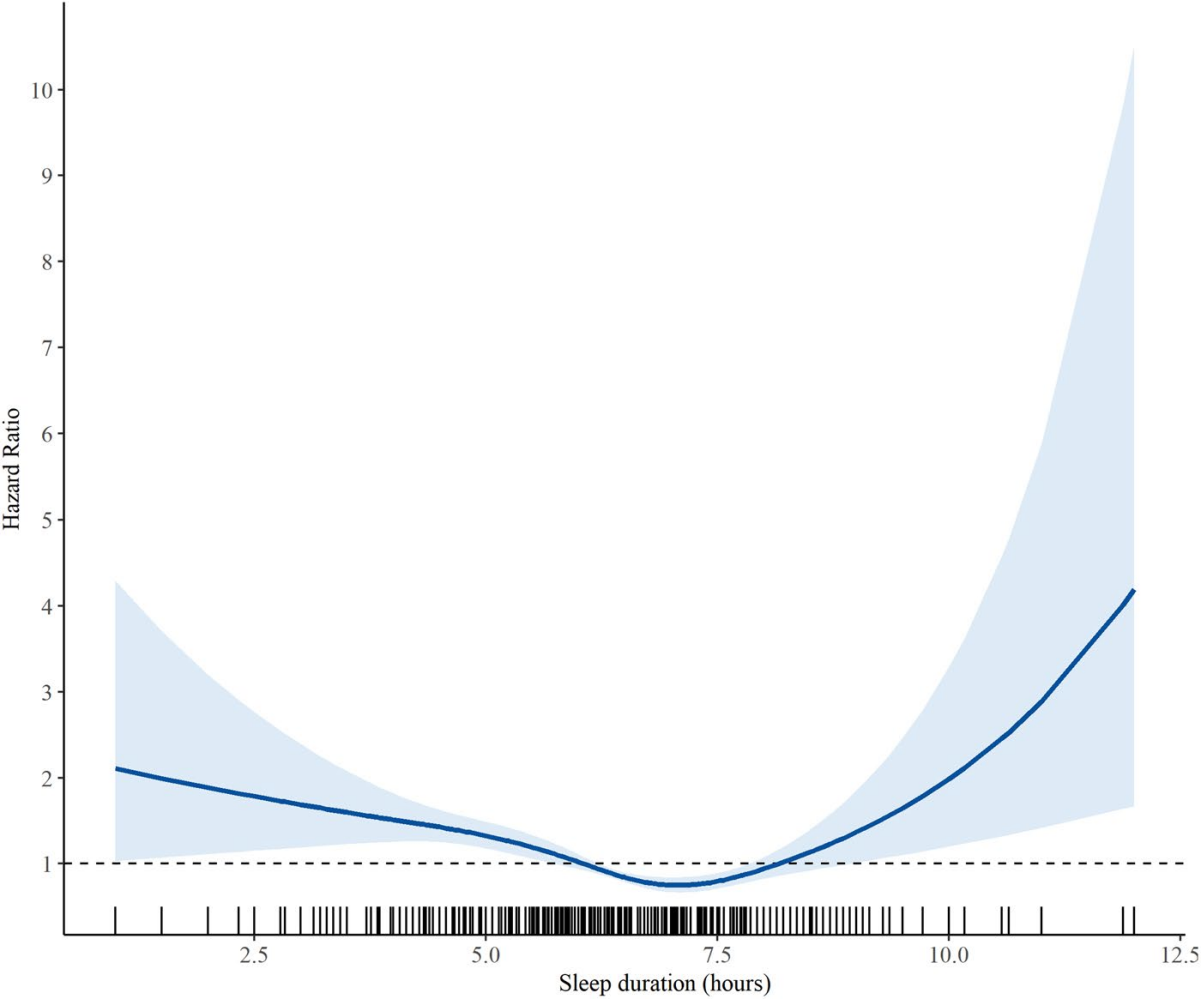
Cox proportional hazard spline curves of incidence CKD based on sleep duration.

Figure 4 presents the spline analysis derived from the crude Cox proportional hazards model. A notable non-linear U-shaped relationship was identified between the onset of new CKD and sleep length. The nadir, or lowest point of this relationship, occurred at 7.071 h, which is within the usual sleep length (7h ≤ to ≤8h).

## 4. Discussion

In this Korean prospective cohort study, results demonstrated a significant association between sleep duration and the new cases of CKD in middle-aged and elderly individuals. Participants who reported either insufficient or excessive sleep durations exhibited a higher incidence of new-onset CKD in contrast to those having standard sleep length, who served as the reference group. Furthermore, age and sex differences were observed in the association between sleep duration and CKD incidence. Importantly, the significant link between insufficient or excessive sleep durations and CKD incidence was observed in individuals aged 40–64 years, suggesting a more pronounced impact in younger participants. Conversely, the lack of a significant association between sleep duration and incident CKD in older adults aged 65 years and above may be attributed to several factors: their generally higher comorbidity burden, which can exert a more profound influence on CKD progression [25], and age-related physiological alterations in sleep patterns [26], both of which can potentially mask the contribution of sleep duration.

The correlation between CKD and excessive sleep length in females, as well as insufficient sleep length in males, has been identified as significant. These sex differences may arise from various pathophysiological mechanisms, including the involvement of the neurotransmitter serotonin, the neurohormone melatonin, and amino acid tryptophan. Furthermore, the results may be influenced by sex-specific differences in the stimulation of peroxisome proliferator-activated receptor α [27]. In females, the heightened risk of CKD observed with prolonged sleep duration may stem from sex-specific hormonal factors (e.g., estrogen, progesterone, and menopausal changes) that impact sleep architecture, thereby potentially aggravating existing CKD risk factors [28]. Elevated testosterone level is linked to lower concentrations of interleukin-6 and CRP [29]. In males, insufficient sleep duration can lead to decreased testosterone levels [30].

Research on obesity and kidney function has consistently shown a correlation between BMI and a heightened risk of CKD [31]. Our findings indicate that the synergistic effect between obesity and prolonged sleep duration suggests that the combination of these two factors may accelerate kidney injury by exacerbating inflammatory processes and metabolic dysregulation [32]. In our findings, the observed lack of a significant association between unhealthy sleep duration and CKD risk in diabetic patients suggests that kidney disease progression, such as diabetic nephropathy, may overwhelm the indirect effects of sleep duration, or that the severe metabolic dysregulation inherent in diabetes could mask sleep’s contribution to CKD risk [33]. The significant association observed in hypertensive patients emphasizes the importance of sleep in mediating cardiovascular and renal health. Unhealthy sleep durations can influence blood pressure regulation and the sympathetic nervous system, thereby accelerating the development of CKD [34]. Additional exploration is required to examine the connection between sleep length and CKD across different clinical contexts, considering the initial disease status.

This research enhances the existing body of knowledge by revealing a U-shaped association between sleep duration and CKD incidence in Korean adults. The findings suggest that middle-aged and elderly Koreans should aim for appropriate sleep durations to mitigate CKD development, consistent with a prior American cross-sectional study that examined NHIS data from individuals aged 20 and older [35]. Nonetheless, there have also been reports of variations in results. A study conducted on middle-aged individuals in Japan found no significant relationship between limited sleep length (5 h) and CKD [36]. Moreover, an additional longitudinal investigation indicated that excessive sleep duration did not correlate with an elevated likelihood of CKD [37]. Research involving Swiss adults demonstrated a direct relationship between sleep length and eGFR [38]. The variations in findings from previous research can contribute to the differences in average sleep length across ethnic groups, such as the relatively short average sleep time observed in the Japanese population [39]. Furthermore, discrepancies may arise from the differing classifications of sleep duration and the selected comparison groups; various studies classify sleep lengths under 4, 5, and 6 h as brief sleep durations. The reference groups have also been defined in multiple ways, including 7–8 h [39], 6–7 h [37], and 6–8 h [40]. Additional investigation is required to explore the fundamental causes of these discrepancies.

The precise mechanism linking prolonged sleep duration to CKD remains unclear. However, investigations suggest that excessive sleep length may be correlated with a higher occurrence of sleep fragmentation [41], obstructive sleep apnoea, snoring, which may negatively impact kidney function [42,43]. The occurrence of CKD associated with excessive sleep length can be attributed to physical inactivity [44]. Investigations examining the connection between insufficient sleep length and CKD indicate that short sleep time may cause heightened sympathetic nervous system activity [45] and promote renal vasoconstriction [46], potentially leading to renal hypertension and CKD [47]. Moreover, both insufficient and excessive sleep durations may lead to CKD through shared mechanisms. For example, inadequate sleep may cause disruptions in the renin-angiotensin-aldosterone pathway, leading to increased filtration in the kidneys and subsequent renal injury [48]. In addition, previous investigations have revealed that insufficient and excessive lengths of sleep are linked to increased levels of circulating CRP [49] and interleukin-6 [50]. Elevated systemic inflammation may ultimately contribute to renal dysfunction [51]. Additionally, individuals who do not get sufficient sleep often experience disrupted circadian rhythms [52], which can interfere with various metabolic processes, such as glucose regulation and the balance of renal electrolytes, potentially leading to CKD [53].

Our study utilized a longitudinal design, which facilitated the exploration of the potential temporal relationship between sleep duration and new-onset CKD among middle-aged and elderly Koreans. We also rigorously identified undiagnosed CKD cases by evaluating proteinuria and calculating eGFR of below 60 mL/min/1.73 m^2^. However, this research has several limitations. First, the reliance on self-reported sleep duration over the past month may introduce recall bias. While objective assessments such as polysomnography offer higher precision, their practicality in large population studies is often constrained. Second, although the KDIGO guidelines’ diagnostic definition of CKD requires two consecutive measurements of eGFR <60 mL/min/1.73 m^2^ or proteinuria ≥1+ at three-month intervals, this study was based on a single measurement, which may lead to misclassification bias. Third, the relatively small sample size in certain extreme categories (e.g., individuals with sleep duration ≥9h) may lead to less precise estimates and limit the generalisability of findings specifically for these extreme groups. Nevertheless, the statistical significance observed in these groups suggests a reliable signal within these categories, and our findings align with the commonly reported U-shaped associations between sleep duration and adverse health outcomes. Fourth, our analysis primarily focused on the association between baseline sleep duration and the risk of new-onset CKD. However, while inadequate sleep duration is often dynamic, it can also manifest as a chronic issue. Future research is thus warranted to provide more comprehensive insights into its dynamic relationship with CKD progression. Last, our data were exclusively derived from the KoGES, which comprises a population solely of Korean descent. As genetic predispositions and environmental factors can vary across populations, subsequent research including diverse populations is therefore necessary to enhance the global generalisability of our findings.

Notwithstanding these constraints, to the best of our awareness, this research represents the first identification of a prospective association between sleep length and incident CKD in a community-residing population of middle-aged and older individuals in Korea. Additionally, this research is grounded in a substantial population cohort with long-term observational data.

## 5. Conclusions

This research identified a relationship between the amount of sleep and CKD among Korean adults. Given the importance of maintaining a sleep duration of 7–8 h/day and its sustainability, promoting effective strategies for sleep optimization, such as behavioural interventions and, when clinically indicated, pharmacological assistance, could help prevent the development of new-onset CKD.

## Data Availability

The data that support the findings of this study are available from the corresponding author upon reasonable request. The data are not publicly available due to privacy or ethical considerations. The KoGES data are available from the Clinical & Omics Data Archive (CODA) [https://coda.nih.go.kr/frt/index.do], with permission from the National Institute of Health at the Korea Disease Control and Prevention Agency (KDCA).
